# Pharmacological postconditioning with tanshinone IIA attenuates myocardial ischemia-reperfusion injury in rats by activating the phosphatidylinositol 3-kinase pathway

**DOI:** 10.3892/etm.2014.1820

**Published:** 2014-07-02

**Authors:** XUN YUAN, SONGBO JING, LINGZHEN WU, LIANGLONG CHEN, JUN FANG

**Affiliations:** Department of Cardiology, Fujian Medical University Union Hospital, Fujian Institute of Coronary Heart Disease, Fuzhou, Fujian 350001, P.R. China

**Keywords:** tanshinone IIA, postconditioning, myocardial infarction, reperfusion injury, phosphatidylinositol 3-kinase, mitochondrial permeability transition

## Abstract

Tanshinone IIA, one of the active ingredients in the Chinese medicine Danshen, is cardioprotective when applied prior to sustained myocardial ischemia. The present study aimed to investigate whether pharmacological postconditioning with tanshinone IIA attenuates myocardial ischemia-reperfusion injury when applied prior to prolonged reperfusion following a sustained ischemia. A total of 88 Sprague-Dawley rats received 30 min myocardial ischemia followed by 5 or 120 min reperfusion. Compared with the ischemia-reperfusion model group, the group that received an intravenous injection of 10 mg/kg tanshinone IIA prior to reperfusion had a reduced myocardial infarct size, higher levels of phospho-Akt and phospho-endothelial nitric oxide synthase and less reduction in the optical density of the mitochondria at 540 nm, indicating that the mitochondrial permeability transition (MPT) was attenuated. The cardioprotective effect conferred by tanshinone IIA was abolished by LY294002, a specific inhibitor of phosphatidylinositol 3-kinase (PI3K). These results demonstrate that tanshinone IIA postconditioning protects the myocardium from ischemia-reperfusion injury through the PI3K/Akt pathway, and the MPT may be also involved in this process.

## Introduction

Reperfusion, as the most important technique for salvaging ischemic myocardium, may also lead to detrimental myocardial ischemia-reperfusion injury (MIRI) (1). Ischemic or pharmacological preconditioning, which is respectively induced by a brief ischemia or the application of bioactive substances prior to a sustained ischemia, has been established as an effective cardioprotective mechanism (2,3). However, ischemic events cannot be anticipated in the clinical setting; therefore, the utility of ischemic and pharmacological preconditioning is notably limited.

Ischemic postconditioning, which is induced by a short series of repetitive cycles of reperfusion and ischemia applied immediately at the onset of prolonged reperfusion, has been reported to be cardioprotective against MIRI. However, in the clinical setting, ischemic postconditioning may be difficult to introduce, i.e., repetitive inflations and deflations of the balloon during primary angioplasty may lead to coronary endothelial damages, plaque rupture and embolic events. Therefore, the concept of pharmacological postconditioning by administering bioactive agents, which mimic the protective effects of ischemic postconditioning, deserves more attention for its feasible, effective and safer characteristics (4,5).

Tanshinone IIA, one of the major effective components in conventional Chinese medicine Danshen, which is derived from the dried root or rhizome of *Salvia miltiorrhiza* Bge., has been widely used in adjunctively treating cardiovascular diseases in China for a long time (6). Previous studies have demonstrated that pharmacological preconditioning with tanshinone IIA may protect the heart from MIRI by reducing myocardial infarct size when applied prior to sustained ischemia in rats (7). Furthermore, the phosphatidylinositol 3-kinase (PI3K)/Akt signaling pathway has been shown to be involved in the cardioprotective effect of ischemia- or pharmacological pre- and postconditioning, including tanshinone preconditioning by inhibiting the opening of the mitochondrial permeability transition pore (mPTP) (4,8,9).

However, it remains unclear whether pharmacological postconditioning with tanshinone IIA is able to attenuate MIRI. Such protection would widen and facilitate the clinical application of tanshinone IIA as an adjunct to the early reperfusion therapy of acute myocardial infarction. Therefore, the present study was designed to examine the hypothesis that tanshinone IIA, applied prior to prolonged reperfusion following a sustained ischemia, may exert a cardioprotective effect against MIRI by activating the PI3K/Akt pathway.

## Methods

### Animals and materials

Male Sprague-Dawley (SD) rats (Shanghai SLAC Laboratory Animal Co., Ltd., Shanghai, China), weighing 250–300 g, were used in this study which conformed to the Guide for the Care and Use of Laboratory Animals published by the US National Institutes of Health (NIH Publication no. 85–23, revised 1996) and was approved by the Experimental Animal Care Committee of Fujian Medical University Union Hospital (Fuzhou, China). All the rats were sedated with 75 mg/kg ketamine and 7.5 mg/kg diazepam intraperitoneally. Sodium tanshinone IIA silate was obtained from Shanghai No.1 Biochemical and Pharmaceutical Co., Ltd. (Shanghai, China). Antibodies for phospho-Akt (p-Akt) and total-Akt (t-Akt) were purchased from Santa Cruz Biotechnology, Inc. (Santa Cruz, CA, USA), and for phospho-endothelial nitric oxide synthase (p-eNOS) and total-eNOS (t-eNOS) were obtained from Cell Signaling Technology, Inc. (Boston, MA, USA). Evan’s blue, triphenyltetrazolium chloride (TTC) and LY294002, a specific inhibitor of PI3K, were purchased from Sigma (St. Louis, MO, USA).

### Experimental procedure

**Figure 1:**
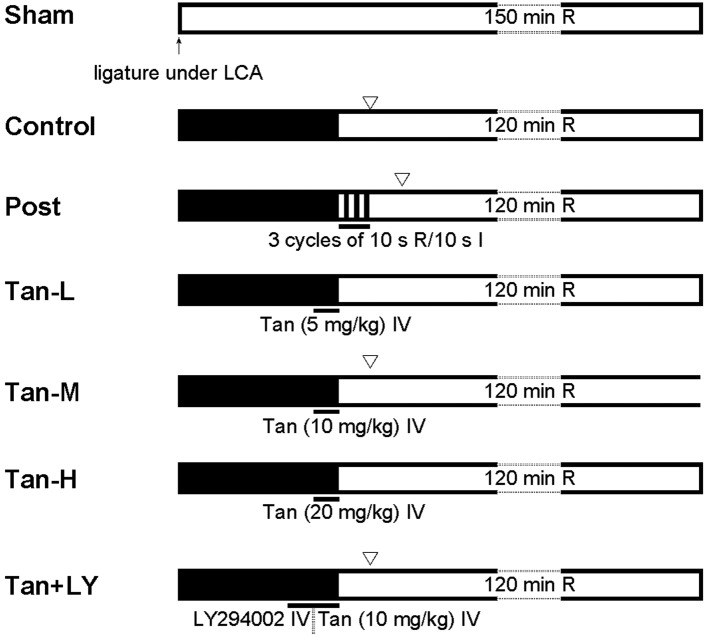
Experimental procedures and animal groups. The rats, exposed to I (dark bar) and R (open bar), were divided into seven groups (n=8 or 16/group): the sham group, subject to surgery but not ligation of the LCA (arrow); the control group, received 30 min I and 5 min or 120 min R; the post group, received 3 cycles of 10 sec R and 10 sec I prior to 120 min R; the tan-L, tan-M and tan-H groups, subject to intravenous injection of 5, 10 and 20 mg/kg of Tan 5 min prior to R, respectively (straight line); the tan+LY group, treated similarly to the Tan-M group, with the addition of an injection 0.3 mg/kg LY, a specific inhibitor of PI3K, 5–10 min prior to R (straight line). In each group, eight rat hearts were harvested following 120 min R. In the control, post, tan-M and tan+LY groups, an additional eight rat hearts were harvested following 5 min R (triangles). Post, ischemic postconditioning; Tan, tanshinone IIA; LY, LY294002; I, ischemia; R, reperfusion; LCA, left main coronary artery.

A total of 88 SD rats were included in the experiment and their left main coronary arteries (LCA) were occluded for 30 min to induce ischemia (I), followed by sustained relaxation for 5 or 120 min to reperfuse (R). All the animals were randomly divided into seven groups (Fig. 1): the sham-surgery group (sham) without ischemia (n=8); the control group (control), receiving I/R without any other intervention (n=16); the ischemic postconditioning group (post), treated the same as the control, with the addition of providing three cycles of 10 sec R and 10 sec I prior to 120 min R (n=16); the low-dose tanshinone group (tan-L), treated the same as the control, with the addition of an intravenous injection of 5 mg/kg tanshinone IIA during 25–30 min I (n=8); the medium-dose tanshinone group (tan-M), treated the same as the control, with the addition of an intravenous injection of 10 mg/kg tanshinone IIA (n=16); the high-dose tanshinone group (tan-H), treated the same as the control, with the addition of receiving an injection of 20 mg/kg tanshinone IIA (n=8); the medium-dose tanshinone plus LY294002 group (tan+LY), treated the same as the tan-M group, with the addition of an intravenous injection of 0.3 mg/kg LY294002, 5–10 min prior to reperfusion (n=16).

### Surgical preparation

The surgical preparations were performed as previously described by Fang *et al* (10). The chest was opened through the fourth intercostal space and a single 7-0 Prolene suture was placed under the LCA, 1–2 mm from its origin. A small polyethylene tube was placed between the ends for reversible coronary artery occlusion. The sham group rats were treated in the same manner, with the exception that the suture was not ligated. At the end of 120 min reperfusion, the left ventricles at risk in eight rats of each group were determined by injection 1 ml 0.1% Evan’s blue after the LCA was ligated again. Cardiac arrest was induced by an intravenous bolus injection of 10% KCl and the left ventricle was transversely sectioned into four slices to assess the infarct size. For the control, post, tan-M and tan+LY groups, an additional eight rats of each group were sacrificed 5 min following reperfusion and the myocardium samples were collected and stored at −80°C for the assessment of mitochondrial permeability transition (MPT), which occurs due to the opening of mPTPs, and western blot examination was performed (Fig. 1).

### Infarct size assessment

The slices of the left ventricle were incubated in a 1% solution of TTC at 36.5°C for 10–15 min. Following this, the slices were fixed in a 10% formaldehyde solution for 24 h. Images of the surfaces that faced the apex were captured by a digital camera. The extents of the blue normal area, plum survival area and the grey area of necrosis were quantified by planimetry using ImageJ 1.36 software from the National Institutes of Health. The area of necrosis (AN), area at risk (AAR; including the grey and red area) and the left ventricular area (LV) were measured. The infarct size was expressed as a percentage of the AAR (AN/AAR) and the risk area was expressed as the AAR/LV.

### Western blot analysis

The samples (60 μg protein/lane) were electrophoresed by 12% sodium dodecyl sulfate polyacrylamide gel electrophoresis, and then electrophoretically transferred onto Hybond-C Extra membranes (Amersham, Pittsburgh, PA, USA). Following treatment with the blocking buffer, the membranes were incubated with primary antibodies (anti-t-Akt and anti-p-Akt; anti-t-eNOS and anti-p-eNOS) and the horseradish peroxidase-conjugated secondary antibody (Sigma). Glyceraldehyde-3-phosphate dehydrogenase (GAPDH) was used as an internal control. The immunoreactive bands were detected by Enhanced Chemiluminescence Plus reagent (Amersham) using an X-ray film (Kodak; Rochester, NY, USA). Target signals were normalized relative to the GAPDH expression and assessed using ImageJ 1.36 software.

### Assessment of mPTP opening

The preparation of mitochondria was adapted from a previously described procedure (11). Isolated mitochondria from cardiomyocytes (1 mg protein) were resuspended in swelling buffer (71 mmol/l sucrose, 215 mmol/l mannitol and 10 mmol/l succinate in 3 mmol/l HEPES, pH 7.4) to a final volume of 2 ml, and incubated at 25°C for 2 min. MPT due to opening of mPTPs, which was induced by 2, 20 and 20 μmol/l CaCl_2_, induced mitochondrial swelling, and was measured spectrophotometrically (DU 800; Beckman Coulter, Inc., Brea, CA, USA) as a reduction in the optical density at 540 nm (OD_540_) in isolated mitochondria (11–13).

### Statistical analysis

Statistical analysis was performed using the SPSS software, version 16.0 (SPSS, Inc., Chicago, IL, USA). All values are expressed as the mean ± standard deviation. The differences among the groups were tested by one-way analysis of variance. When a statistical difference was identified, the least significant difference procedure was applied. P<0.05 was considered to indicate a statistically significant result.

## Results

### Infarct size

**Figure 2:**
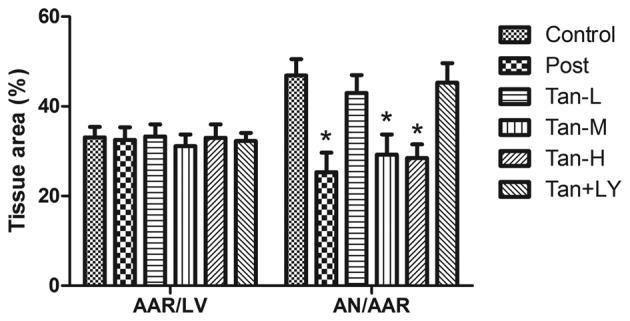
Effects of various treatments on infarct size. AAR is expressed as a percentage of the LV (AAR/LV) and AN as a percentage of the AAR (AN/AAR). Compared with the control group, the post, tan-M, tan-H groups had a significantly reduced infarct size (AN/AAR). The reduction in infarct size induced by 10 mg/kg tanshinone IIA postconditioning was abrogated completely by LY, a specific inhibitor of PI3K. All values are expressed as the mean ± standard deviation (%; n=8/group). ^*^P<0.01 vs. the control, tan-L and tan+LY groups. Post, ischemic postconditioning; tan-L, low-dose tanshinone IIA (5 mg/kg); tan-M, medium-dose tanshinone IIA (10 mg/kg); tan-H, high-dose tanshinone IIA (20 mg/kg); LY, LY294002; AAR, area at risk; LV, left ventricular area; AN, area of necrosis.

No ischemic and necrotic areas were found in the sham-surgery group. No significant difference was identified among the other groups in AAR/LV (F=0.737, P=0.600). Infarct size (AN/AAR) in the post, tan-M and tan-H groups was significantly smaller than that in the control group (25.3±4.3, 29.2±4.5 and 28.5±3.0 vs. 46.9±3.6%, respectively; P<0.01). No significant differences were observed between the control group and the tan-L group (46.9±3.6 vs. 43.0±4.0%), and among the post, medium- and high-dose tanshinone IIA groups. The reduction in infarct size induced in the tan-M group was abrogated completely by LY294002 treatment (29.2±4.5 vs. 45.3±4.3%; P<0.01) and the infarct size following LY294002 treatment was comparable with that of the control group (Fig. 2).

### Expression levels of p-Akt and p-eNOS in the myocardium

**Figure 3:**
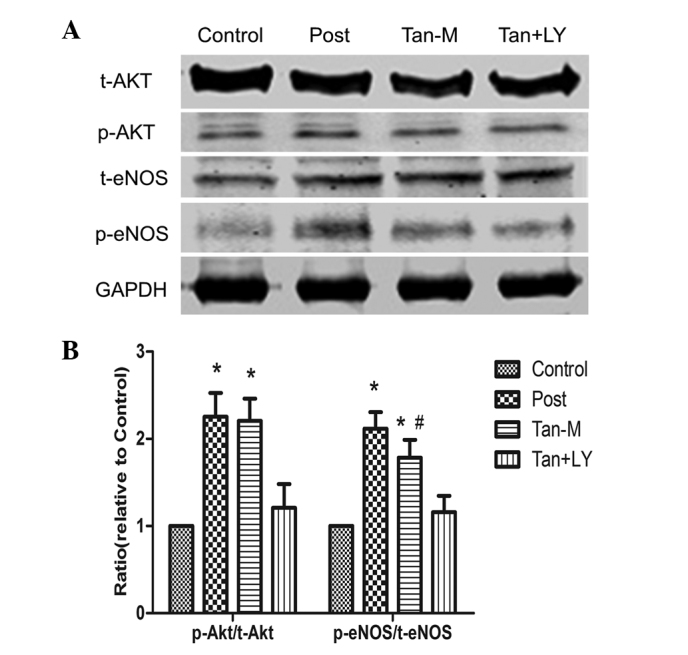
Effects of various treatments on protein expression of p-Akt and p-eNOS. (A) Representative graphs for Akt and eNOS protein expression by western blotting. (B) Quantitative western blot analysis demonstrated the phosphorylation level of Akt and eNOS (relative to the control). Compared with the control group, the expression of p-Akt/t-Akt and p-eNOS/t-eNOS were increased in the post and tan-M groups. The phosphorylation afforded by 10 mg/kg tanshinone IIA postconditioning was abrogated by LY. ^*^P<0.01 vs. the control and tan+LY groups; ^#^P<0.01 vs. the post group. Post, ischemic postconditioning; tan-M, medium-dose tanshinone IIA (10 mg/kg); LY, LY294002; eNOS, endothelial nitric oxide synthase; p, phospho; t, total.

Compared with the control group, the post and tan-M groups had higher p-Akt/t-Akt and p-eNOS/t-eNOS expression ratios (p-Akt/t-Akt, 1.0±0.0 vs. 2.3±0.3 and 2.2±0.3; p-eNOS/t-eNOS, 1.0±0.0 vs. 2.1±0.2 and 1.8±0.2, respectively; all P<0.01). No statistically significant difference was observed in the p-Akt/t-Akt ratio between the post and tan-M groups; however, the p-eNOS/t-eNOS ratio in the tan-M group was lower than that in the post group (P<0.01). Compared with the phosphorylation level in the tan-M group, that in the LY294002 group was significantly lower (p-Akt/t-Akt, 2.2±0.3 vs. 1.2±0.3; P<0.01; p-eNOS/t-eNOS, 1.8±0.2 vs. 1.2±0.2, P<0.01; Fig. 3).

### Ca^2+^ induced mPTP opening

**Figure 4:**
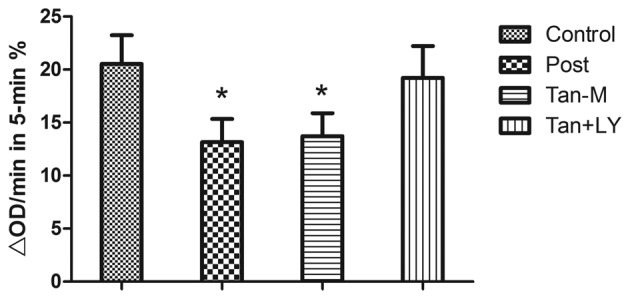
Effects of various treatments on MPT. MPT was expressed as a reduction in OD_540_ during 5 min (ΔOD/min). In the post and tan-M groups, ΔOD/min was lower than that in the control group. The reduction in MPT induced by 10 mg/kg tanshinone IIA postconditioning was eliminated by LY. ^*^P<0.01 vs. the control and tan+LY groups. MPT, mitochondrial permeability transition; post, ischemic postconditioning; tan-M, medium-dose tanshinone IIA (10 mg/kg); LY, LY294002; OD_540_, optical density at 540 nm.

MPT was expressed as a reduction in OD_540_ during 5 min (ΔOD/min). In the post and tan-M groups, ΔOD/min was lower than that in the control group (13.2±2.2 and 13.7±2.2 vs. 20.5±2.7%, respectively; P<0.01). No statistically significant difference was observed between the post and tan-M groups. The reduction in MPT afforded by the Tan-M group was eliminated by LY294002 treatment (13.7±2.2 vs. 19.2±3.0%; P<0.01; Fig. 4).

## Discussion

In the present study, it was demonstrated that pharmacological postconditioning with tanshinone IIA, one of the active ingredients in the Chinese medicine Danshen, protects the myocardium from ischemia-reperfusion injury by activating the PI3K/Akt-eNOS pathway, and the blockage of mPTP opening may be involved in this cardioprotective effect.

In animal models, a variety of pharmacological treatment strategies have been demonstrated to reduce infarct size by activating the reperfusion injury salvage kinase (RISK) pathway, which includes PI3K/Akt and extra-cellular signal-regulated protein kinase 1/2 pathways, when applied at the onset of reperfusion (14). However, a number of these strategies have produced disappointing results when translated into the clinical setting (15). This may be as a result of the marked differences that exist between the clinical setting and studies in animal models. However, the pharmacological agents applied in these studies are also important factors, which may lead to negative clinical results. Therefore, studies investigating more clinically feasible and effective pharmacological strategies are required to confer cardioprotection by activating pro-survival kinases, serving as an adjunct to early reperfusion therapy for acute myocardial infarction.

Tanshinone IIA, a key component of the Chinese medicine Danshen obtained from *Salvia miltiorrhiza*, has been used in clinical practice for cardiovascular and cerebrovascular diseases in Asia. A number of studies have demonstrated that tanshinone IIA protects cardiac myocytes against oxidative stress-triggered damage and apoptosis (16), prevents myocardial hypertrophy (17), protects against sudden cardiac death (18) and attenuates myocardial ischemic (19) and reperfusion injury (20). However, the majority of studies (7,20) have focused on applying tanshinone IIA prior to ischemia, which limits its use in clinical practice. In the present study, the clinical application of tanshinone IIA was widened, as the data obtained demonstrate that when applied prior to prolonged reperfusion following sustained ischemia, tanshinone IIA at both medium and high doses, which have been used in other studies for cardioprotection, resulted in a reduction in infarct size comparable to that of ischemic postconditioning. In the present study, three dosages (5, 10 and 20 mg/kg) were utilized. Since low-dose tanshinone IIA failed to reduce the infarct size, and medium-dose tanshinone IIA elicited a similar cardioprotective effect in infarct size reduction as high-dose tanshinone IIA, the dosage of 10 mg/kg tanshinone IIA may serve as a more cost-effective optimal dose and was used to further examine the molecular mechanisms involved in this process.

Several studies have demonstrated that the activation of the RISK pathway is involved in cardioprotection in animal models (14). The RISK pathway, first reported by Hausenloy and Yellon, is one of crucial mechanisms in the regulation of cell survival (14). eNOS is an important downstream target of PI3K/Akt (21). Previous studies have demonstrated that the mPTP, a non-specific pore associated with contact sites between the outer and inner mitochondrial membranes, is a key end-effector of reperfusion injury; its opening may lead to MPT, ultimately causing severe apoptosis and cell necrosis (22).

The present study also investigated whether tanshinone IIA attenuates ischemia-reperfusion injury through the PI3K/Akt/eNOS-mPTP pathway. The results demonstrated that at 5 min reperfusion, the myocardial protein levels of p-Akt/t-Akt and p-eNOS/t-eNOS were comparably elevated in ischemic and tanshinone IIA postconditioning groups, as compared with those in the control group, suggesting that Akt phosphorylation may be activated by the two postconditioning methods at the early reperfusion. Furthermore, a specific PI3K inhibitor, LY294002, was used to reduce Akt and its downstream eNOS phosphorylation levels, which eliminated the cardioprotection induced by tanshinone IIA. This demonstrated that tanshinone IIA confers cardioprotection through the PI3K/Akt/eNOS pathway when applied prior to reperfusion following sustained ischemia. It was also identified that p-eNOS/t-eNOS in the tan-L group was lower than that in the post group, which indicates that tanshinone IIA may also activate other downstream signaling proteins (not only eNOS) of PI3K to confer cardioprotection. In addition, MPT was detected at 5 min reperfusion, the same time point when pro-survival signal kinases were upregulated by tanshinone IIA postconditioning. The results demonstrated that ischemic and tanshinone IIA postconditioning both lowered the ΔOD/min, indicating attenuated mitochondrial swelling. However, these effects were blocked by LY294002, which suggested that inhibition of mPTP opening by tanshinone IIA postconditioning may be regulated by the PI3K/Akt pathway.

Notably, a number of studies (8,14,23,24) have indicated that the PI3K/Akt pathway is involved in the cardioprotective effect afforded by ischemia- or pharmacological pre- and postconditioning by inhibiting mPTP opening. One previous study has also revealed that pretreatment with tanshinone IIA may inhibit mPTP opening dose-dependently, and its cardioprotective effect may be via the inhibition of pore opening during reperfusion (25). More recently, Zhang *et al* (20) reported that tanshinone IIA pretreatment elicits cardioprotection in diabetic rats via the PI3K/Akt-dependent pathway. The results from the present study are consistent with these previous findings.

There are several limitations to consider in the present study. Firstly, specific mPTP openers or inhibitors were not used to confirm the causal correlation between tanshinone postconditioning and mPTP opening. Secondly, further studies are required to determine the efficacy of tanshinone postconditioning in clinical practice.

The present study demonstrated that pharmacological postconditioning with tanshinone IIA protects the rat myocardium from ischemia-reperfusion injury through the PI3K/Akt-eNOS pathway, and the blockage of MPT may have an important role in this process. Since tanshinone IIA has been safely used in clinical practice, particularly in patients with ischemic heart diseases in Asia, the concept of tanshinone postconditioning by the PI3K/Akt-mPTP pathway may represent a practical solution to reduce ischemia-reperfusion injury as an adjuvant to current reperfusion strategies.
